# Long Chain Omega-3 Polyunsaturated Fatty Acid Supplementation Alleviates Doxorubicin-Induced Depressive-Like Behaviors and Neurotoxicity in Rats: Involvement of Oxidative Stress and Neuroinflammation

**DOI:** 10.3390/nu8040243

**Published:** 2016-04-23

**Authors:** Yan-Qin Wu, Rui-Li Dang, Mi-Mi Tang, Hua-Lin Cai, Huan-De Li, De-Hua Liao, Xin He, Ling-Juan Cao, Ying Xue, Pei Jiang

**Affiliations:** 1 Institute of Clinical Pharmacy & Pharmacology, Second Xiangya Hospital, Central South University, Changsha 410011, China; wuyanqin1992@163.com (Y.-Q.W.); tangmimi1989@163.com (M.-M.T.); ghostspecialist@163.com (H.-L.C.); hexin526@126.com (X.H.); caolingjuan@126.com (L.-J.C.); xueying091@126.com (Y.X.); 2 Department of Pharmacy, Jining First People’s Hospital, Jining Medical University, Jining 272000, China; ruilidang@gmail.com; 3 Department of Pharmacy, Hunan Cancer Hospital, Central South University, Changsha 410011, China; liaodehua1125@126.com

**Keywords:** depression, neurotoxicity, ω-3 PUFAs, doxorubicin, oxidative stress, neuroinflammation

## Abstract

Doxorubicin (DOX) is a chemotherapeutic agent widely used in human malignancies. Its long-term use can cause neurobiological side-effects associated with depression. Omega-3 polyunsaturated fatty acids (ω-3 PUFAs), the essential fatty acids found in fish oil, possess neuroprotecitve and antidepressant activities. Thus, the aim of this study was to explore the potential protective effects of ω-3 PUFAs against DOX-induced behavioral changes and neurotoxicity. ω-3 PUFAs were given daily by gavage (1.5 g/kg) over three weeks starting seven days before DOX administration (2.5 mg/kg). Open-field test (OFT) and forced swimming test (FST) were conducted to assess exploratory activity and despair behavior, respectively. Our data showed that ω-3 PUFAs supplementation significantly mitigated the behavioral changes induced by DOX. ω-3 PUFAs pretreatment also alleviated the DOX-induced neural apoptosis. Meanwhile, ω-3 PUFAs treatment ameliorated DOX-induced oxidative stress in the prefrontal cortex and hippocampus. Additionally, gene expression of pro-inflammatory cytokines, including IL-1β, IL-6, and TNF-α, and the protein levels of NF-κB and iNOS were significantly increased in brain tissues of DOX-treated group, whereas ω-3 PUFAs supplementation significantly attenuated DOX-induced neuroinflammation. In conclusion, ω-3 PUFAs can effectively protect against DOX-induced depressive-like behaviors, and the mechanisms underlying the neuroprotective effect are potentially associated with its anti-oxidant, anti-inflammatory, and anti-apoptotic properties.

## 1. Introduction

Doxorubicin (DOX), a quinone-containing antitumor drug, is widely used for the treatment of cancer, especially for treating breast and esophageal carcinomas [1]. However, it has been noted that long-term use of DOX tends to induce neurotoxicity and may cause neuropsychiatric diseases including depression, anxiety, and impaired cognition function [2,3,4]. Previous studies of patients undergoing chemotherapy for breast cancer have consistently shown their depressed mood and decreased interest in surroundings [5], which highlight the importance of further understanding on the neurotoxic effects of DOX and seeking potential therapeutic strategies.

The impairment of neurogenesis and increased neural apoptosis in the limbic brain regions, including the prefrontal cortex and hippocampus, is considered as one of the leading causes of depression. It was reported that DOX-mediated generation of free radicals in the brain tissues increases lipid peroxidation and protein oxidation, and alters the antioxidant defense system, eventually leading to neuropsychological changes [3,6]. Moreover, increased generation of superoxide anions induced by DOX may elevate the level of circulating tumor necrosis factor-alpha (TNF-α) which can directly pass blood brain barrier (BBB), and activate glial cells to initiate the local production of pro-inflammatory cytokines which exacerbate the oxidative stress and neural apoptosis [1,7,8]. In addition, many inflammatory mediators such as TNF-α and nuclear factor-kappa B (NF-κB) have been shown to be critically involved in neuroinflammation both in animal models and in patients undergoing chemotherapy [9,10]. Therefore, in the treatment of DOX-induced depression, an approach involving reduced production of reactive oxygen species (ROS) and inhibited release of neurotoxic pro-inflammatory mediators might be beneficial.

Among the potent approaches, recent studies highlight the effectiveness of ω-3 polyunsaturated fatty acids (ω-3 PUFAs) supplementation as a potential treatment strategy for brain damage [11,12,13,14]. Dietary intake of ω-3 PUFAs, such as eicosapentaenoic acid (EPA, 20:5, ω-3) and docosahexaenoic acid (DHA, 22:6, ω-3), is known to be beneficial for mental health [15]. Many pharmacological studies demonstrated that EPA and DHA possess chemopreventive and antidepressant activities [16,17,18,19]. These effects have been mainly attributed to their anti-oxidant and anti-inflammatory properties, which had been described in a variety of models [20,21,22]. ω-3 PUFAs were shown to lead to a decrease in lipid peroxidation and suppress oxidative stress in brain tissues [23,24]. Additionally, Irene *et al*. (2014) showed that DHA suppresses production of inflammatory mediators, such as NF-κB, TNF-α, and iNOS in a mouse model of spinal cord injury [12]. In addition, another study found that ω-3 PUFAs pretreatment could effectively protect the testicular cells from DOX-induced apoptotic injury, suggesting the anti-apoptotic activity of ω-3 PUFAs [17].

Based on the above findings, the aim of this study was to investigate the potential protective effects of ω-3 PUFAs against DOX-induced neurotoxicity and depression-like behaviors in rats. In addition, the possible underlying mechanisms, including anti-oxidant, anti-inflammatory, as well as anti-apoptotic effects of ω-3 PUFAs in brain tissues, were investigated.

## 2. Materials and Methods

### 2.1. Animals

Sprague-Dawley rats (Male, 150–180 g; the Experimental Animal Center of the Second Xiangya Hospital) were initially housed in groups in a temperature-controlled environment under a 12/12 h light/dark cycle with free access to food and water except during experimental procedures. This study was approved by the Animal Care and Use Committee of Central South University (protocol number 036/2015). All experiments were performed in accordance with the Guide for Care and Use of Laboratory Animals (Chinese Council).

### 2.2. Experimental Design

Animals were divided randomly into four groups (*n* = 8): (1) control; (2) PUFA; (3) DOX; and (4) DOX + PUFA. The untreated control group was injected with the appropriate volume of the normal saline. Rats in PUFA group received daily gavage of 1.5 g/kg ω-3 PUFAs (EPA 34%, DHA 24%, Sheng Tianyu Biotechnology, Wuhan, China) for three weeks, serving as another control group to exclude any toxic effects. The DOX group was given DOX (Zhejiang Hisun Pharmaceutical Company Limited, Taizhou, China) every two days for a total of seven injections via intraperitoneal injection at a dose of 2.5 mg/kg. The DOX + PUFA group received ω-3 PUFAs daily for three weeks starting one week before giving DOX. The doses of ω-3 PUFAs and DOX were based on previous studies [1,15]. The body weight of these rats was monitored throughout the experiment, and drug doses were adjusted accordingly.

At the end of the three weeks, behavioral tests were carried out and the rats were anesthetized with 10% chloral hydrate (4 mL/kg) [15]. Blood was collected and the brain was quickly removed after cardiac perfusion with phosphate-buffered saline (PBS) (pH = 7.2). The left hemisphere of the brain was maintained in 4% paraformaldehyde and then embedded in paraffin, prepared for histopathological examination and immunohistochemical staining. For the right hemisphere, the prefrontal cortex and hippocampus were dissected and used for oxidative stress measurement and Western blot and PCR analysis.

### 2.3. Behavioral Test

#### 2.3.1. Open-Field Test (OFT)

The test was performed in a square arena (90 cm × 90 cm × 40 cm) with the floor divided into 25 equal squares by black lines. The rat was placed into the center of the open field and allowed to move freely over a 5-min period. The apparatus was cleaned with 75% ethanol prior to each test session to eliminate odors. Behavioral parameters to assess locomotor activity and exploratory behavior (latency period, number of crossings, and rearing) were recorded.

#### 2.3.2. Forced Swimming Test (FST)

FST is widely employed to screen antidepressant efficacy and depressive-like behavior in rodents. The test was performed as previously described [15]. In brief, each rat was placed in a plastic drum (45 cm height, 25 cm diameter) containing approximately 35 cm of water (24 ± 1 °C) for a 15-min pretest. After swimming, rats were dried with towels and placed back in their home cage. 24 h later, the rat was exposed to the same experimental conditions outlined above for a 5-min FST. The measured parameters were immobility, swimming and struggling scores. Water was changed before each trial. Immobility was defined as floating passively and only making slight movements to keep the head above water. Each test session was videotaped and the duration of immobility was scored by an experienced observer blind to the experiment design.

### 2.4. Measurement of Oxidative Stress

The prefrontal cortex and hippocampus were homogenized using Precellys 24 multifunctional homogenizer (Bertin Technologies, Aix-en-Provence, France). An aliquot of the homogenate was used for the assay of malondialdehyde (MDA) formation and superoxide dismutase (SOD) activity. MDA levels and SOD activity were determined using the Lipid Peroxidation MDA Assay Kit and Total Superoxide Dismutase Assay Kit with WST-1 (Nanjing Jiancheng Bioengineering Institute, Nanjing, China) respectively, following the manufacturer’s instructions.

### 2.5. Western Blot Analysis

For Western blot analysis, total protein was prepared from the prefrontal cortex and hippocampus, and the concentration was determined using Bradford method. Samples were loaded on a precast 12% SDS-PAGE gel with 10 μg protein in each lane. Proteins in the gels were transferred to a PVDF membrane and blocked for 1 h in 5% non-fat dry milk in TBS-T (25 mM Tris, pH 7.5, 150 mM NaCl, 0.05% Tween-20). The following antibodies and concentrations were used over night at 4 °C; iNOS (Proteintech; 1:500), NF-κB (Proteintech; 1:500), and β-actin (Proteintech; 1:4000). It was then probed with HRP-conjugated secondary antibody for 40 min. The film signal was digitally scanned and then quantified using Image J software (National Institutes of Health, Bethesda, MD, USA). The signals were normalized to β-actin as an internal standard.

### 2.6. Real-Time PCR Analysis

Total RNA was extracted from the prefrontal cortex and the hippocampus using Trizol reagent (Invitrogen Corp., Carlsbad, CA, USA) following the manufacturer’s instructions for detection of the gene expression of IL-1β, IL-6, TNF-α, Bax, Bcl-2, and Bcl-xl. RNA concentration was determined for quantity and integrity using the spectrophotometry (Jingke, Ningbo, China). cDNA was produced using Revert Aid First Strand cDNA Synthesis Kit (Thermo Fisher Scientific, Tewksbury, MA, USA). Quantitative PCR was performed on Bio-rad Cx96 Detection System (Bio-rad, Hercules, CA, USA) using SYBR green PCR kit (Applied Biosystems Inc., Woburn, MA, USA) and gene-specific primers. A 5 ng cDNA sample was used with 40 cycles of amplication. Each cDNA was tested in triplicate. Relative quantitation for PCR product was normalized to β-actin as an internal standard. The sequences of gene-specific primers are listed in Table 1.

### 2.7. Histopathological Examination

For light microscopy, autopsy samples were taken from the brain of rats in different groups and embedded in paraffin. The paraffin tissue blocks were prepared for sectioning at 5 micron thickness by sledge microtome. The obtained tissue sections were collected on glass slides, deparaffinized, stained by hematoxylin and eosin stain for routine examination, and then examination was done through the light electric microscope.

### 2.8. Immunohistochemical Study

Neurocyte apoptosis was evaluated by terminal deoxynucleotidyl transferase-mediated deoxyuridine triphosphate nick end labeling (TUNEL) assay. The TUNEL method, which detects fragmentation of DNA in the nucleus during apoptotic cell death *in situ*, was employed using an apoptosis detection kit (Keygen Biotech, Nanjing, China). The average ratio of the total TUNEL-positive neurocyte number was calculated from randomly selected 10 microscopic high-power fields for each rat in all of the groups. This ratio represented the apoptotic index of the sample and was compared between groups.

### 2.9. Statistical Analysis

All statistical procedures were performed on Statistical Package for the Social Science (SPSS) version 18 (SPSS Inc., Chicago, IL, USA). Data were expressed as mean ± SD, and analyzed statistically by one-way analysis of variance (ANOVA) with Tukey *post hoc* multiple comparisons. The prior level of significance was established at *p* < 0.05.

## 3. Results

### 3.1. Effect of DOX and ω-3 PUFAs on Body Weight Gain and Behavioral Changes

**Figure 1:**
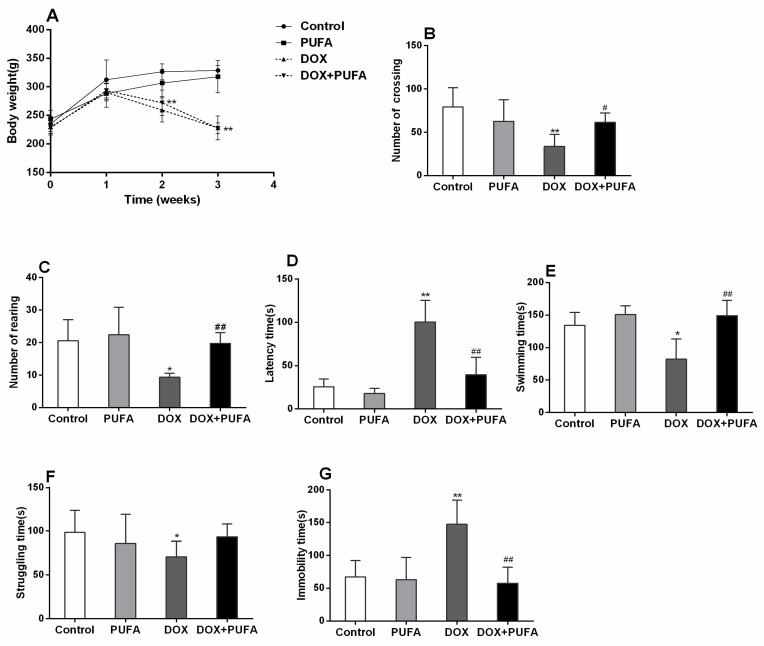
Body weight gain and behavioral test. Effect of DOX and ω-3 PUFAs on body weight gain (**A**) and open field test: numbers of crossing (**B**); number of rearing (**C**); and latency time (**D**); effect of DOX and ω-3 PUFAs on forced swimming test: swimming time (**E**); struggling time (**F**); and immobility time (**G**). Data are expressed as means ± SD (*n* = 6–7). * *p* < 0.05, ** *p* < 0.01 compared to control group. ^#^ *p* < 0.05, ^##^ *p* < 0.01 compared to DOX-injected group.

As shown in Figure 1A, DOX-treated rats showed significantly decreased body weight gain when compared to control animals (Figure 1A, *p* < 0.01), whereas ω-3 PUFAs pretreatment had no influence on the body weight gain in both DOX or vehicle treated rats, which is consistent with the results of the previous studies [15,25,26]. The numbers of crossings and rearing, as well as latency time, were observed in the OFT. The DOX group exhibited a significant decrease in numbers of crossings (Figure 1B, *p* < 0.01) and rearing (Figure 1C, *p* < 0.05), as well as a significantly increased latency time (Figure 1D, *p* < 0.01) when compared to control group. Pretreatment with ω-3 PUFAs significantly increased the numbers of crossings (Figure 1B, *p* < 0.05) and rearing (Figure 1C, *p* < 0.01), and markedly decreased latency time (Figure 1D, *p* < 0.01) in DOX-treated rats, showing more locomotor activity and exploratory behavior. In the FST, two weeks of DOX administration led to a significant decrease in the swimming (Figure 1E, *p* < 0.05) and struggling (Figure 1F, *p* < 0.05) times as well as a significant increase in the immobility time (Figure 1G, *p* < 0.01) when compared to the control group. On the other hand, ω-3 PUFAs mitigated the behavioral changes in FST, except that the struggling time in the ω-3 PUFAs pretreated group was slightly, but not significantly, increased as compared with DOX-treated group.

### 3.2. Effects of DOX and ω-3 PUFAs on Oxidative Stress Markers

**Figure 2:**
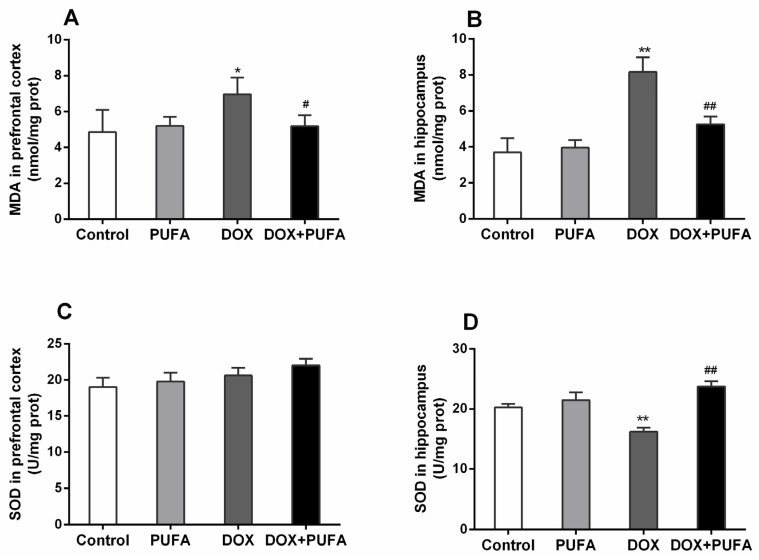
Effects of DOX and ω-3 PUFAs on MDA and SOD in the prefrontal cortex and hippocampus. Data are expressed as means ± SD (*n* = 6–7). * *p* < 0.05, ** *p* < 0.01 compared to control group. ^#^ *p* < 0.05, ^##^ *p* < 0.01 compared to the DOX-injected group.

In the DOX-treated rats, MDA level was significantly enhanced both in the prefrontal cortex (Figure 2A, *p* < 0.05) and hippocampus (Figure 2B, *p* < 0.01), while the SOD level was significantly reduced only in the hippocampus (Figure 2D, *p* < 0.01) with no remarkable change in the prefrontal cortex (Figure 2C). On the other hand, ω-3 PUFAs supplementation significantly ameliorated the changes of SOD and MDA levels in the DOX-treated group with a significant increase of SOD level (Figure 2D, *p* < 0.01) in the hippocampus and a significant decrease of MDA level both in the prefrontal cortex (Figure 2A, *p* < 0.05) and hippocampus (Figure 2B, *p* < 0.01), showing the protective effect of ω-3 PUFAs against DOX-induced oxidative stress in brain tissues.

### 3.3. Effects of DOX and ω-3 PUFAs on Neuroinflammation Biomarkers

**Figure 3:**
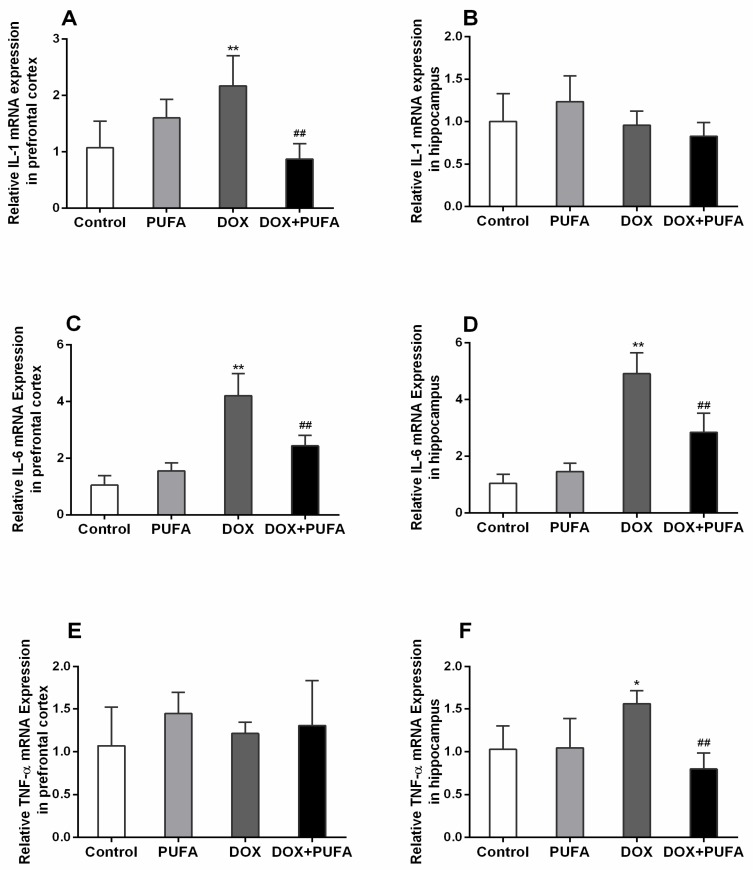
Effects of DOX and ω-3 PUFAs on gene expression of IL-1, IL-6, and TNF-α in the prefrontal cortex and hippocampus. Data are expressed as means ± SD (*n* = 6–7). * *p* < 0.05, ** *p* < 0.01 compared to control group. ^#^ *p* < 0.05, ^##^ *p* < 0.01 compared to the DOX-injected group.

The DOX group showed a significant increase in gene expressions of IL-1β (Figure 3A, *p* < 0.01) and IL-6 (Figure 3C, *p* < 0.01) in the prefrontal cortex, while in the hippocampus, gene expressions of IL-6 (Figure 3D, *p* < 0.01) and TNF-α (Figure 3F, *p* < 0.05) were remarkably enhanced as compared with control group. However, these elevated gene expressions were significantly attenuated by ω-3 PUFAs supplementation. The DOX + PUFA group showed significantly decreased gene expressions of IL-1β (Figure 3A, *p* < 0.01) and IL-6 (Figure 3C, *p* < 0.01) in the prefrontal cortex, and reduced gene expressions of IL-6 (Figure 3D, *p* < 0.01) and TNF-α (Figure 3F, *p* < 0.01) in the hippocampus when compared to DOX group.

**Figure 4:**
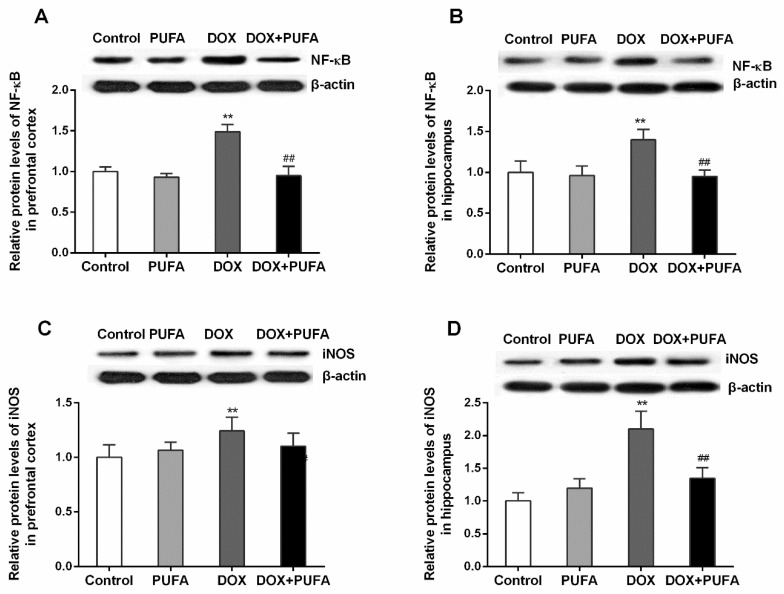
Effects of DOX and ω-3 PUFAs on protein expression of NF-κB and iNOS in the prefrontal cortex and hippocampus. Data are expressed as means ± SD (*n* = 6–7). * *p* < 0.05, ** *p* < 0.01 compared to control group. ^#^ *p* < 0.05, ^##^ *p* < 0.01 compared to the DOX-injected group.

In the prefrontal cortex and the hippocampus of DOX-treated rats, the protein expression of NF-κB (Figure 4A,B; *p* < 0.01, both) and iNOS (Figure 4C,D; *p* < 0.01, both) was markedly increased as compared to the vehicle-treated rats. In correspondence to the modulating effects of ω-3 PUFAs on the inflammatory cytokines, ω-3 PUFAs downregulated protein level of NF-κB (Figure 4A,B; *p* < 0.01, both) and iNOS (Figure 4D; *p* < 0.01) when compared to the animals treated with DOX alone.

### 3.4. Effects of DOX and ω-3 PUFAs on Histopathological Changes

**Figure 5:**
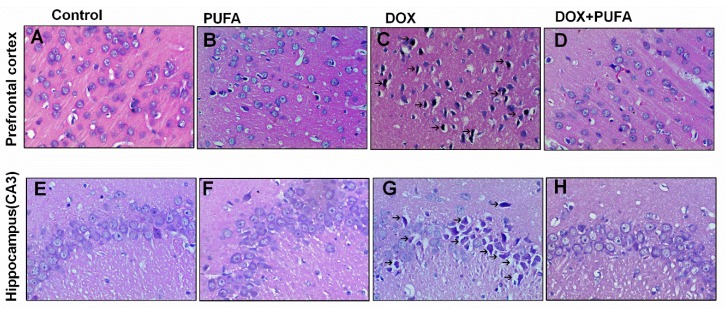
Effects of DOX and ω-3 PUFAs on histological changes in the prefrontal cortex and hippocampus (magnification 400×). Note the presence of degenerating neurons (arrows) in the DOX-treated group.

Histopathological alterations in brain specimens from different treated groups are shown in Figure 5. Sections from the control group showed normal histology (Figure 5A,E). DOX-administered brain tissues showed more frequent nuclear pyknosis (Figure 5C,G), whereas rats pretreated with ω-3 PUFAs showed a reduced number of nuclear pyknosis and almost usual architecture similar to those of the normal brain tissues (Figure 5D,H).

### 3.5. Effects of DOX and ω-3 PUFAs on Neural Apoptotic Markers

**Figure 6:**
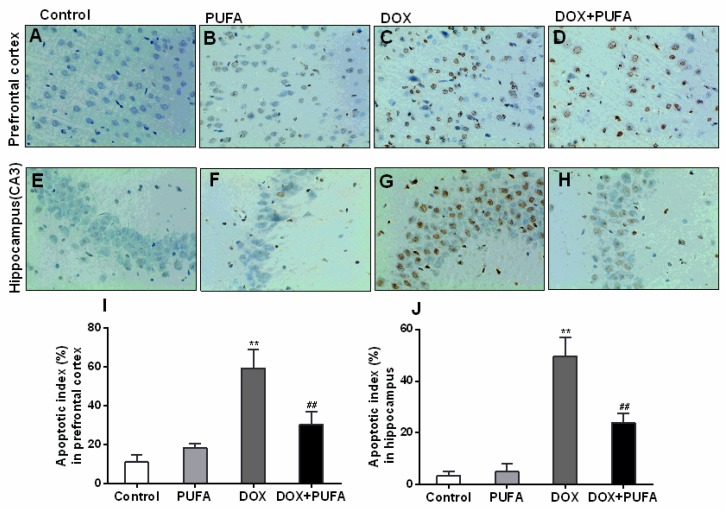
Effects of DOX and ω-3 PUFAs on TUNEL-positive cells in the prefrontal cortex and hippocampus. (**A**)–(**H**) show the TUNEL-staining positive cells through the light electric microscope followed by observation at 400×; (**I**) and (**J**) show the quantitative analysis of TUNEL results in the prefrontal cortex and hippocampus. Data are expressed as means ± SD (*n* = 6–7). * *p* < 0.05, ** *p* < 0.01 compared to control group. ^#^ *p* < 0.05, ^##^ *p* < 0.01 compared to the DOX-injected group.

As revealed in Figure 6, after two weeks of treatment, brain tissues exposed to DOX contained much more TUNEL-positive cells (Figure 6C,G) in contrast to those pretreated with ω-3 PUFAs (Figure 6D,H), indicating the pro-apoptotic effects of DOX and anti-apoptotic effects of ω-3 PUFAs in brain tissues. Figure 6I,J show the apoptotic index in the prefrontal cortex and hippocampus of rats in different groups. The percentage of apoptotic neurocytes was significantly increased by DOX (*p* < 0.01), but was partly restored by ω-3 PUFAs pretreatment (*p* < 0.01).

**Figure 7:**
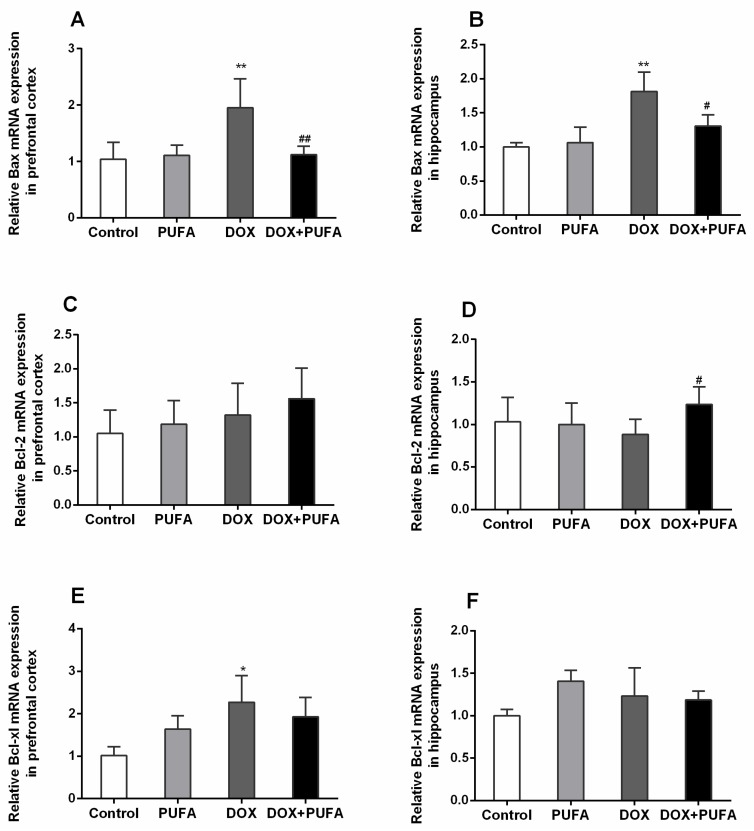
Effects of DOX and ω-3 PUFAs on gene expression of Bax, Bcl-2, and Bcl-xl in the prefrontal cortex and hippocampus. Data are expressed as means ± SD (*n* = 6–7). * *p* < 0.05, ** *p* < 0.01 compared to the control group. ^#^ *p* < 0.05, ^##^ *p* < 0.01 compared to the DOX-injected group.

Consistent with the results of the TUNEL staining, a significant increase in gene expression of pro-apoptotic Bax was observed both in the prefrontal cortex (Figure 7A, *p* < 0.01) and the hippocampus (Figure 7B, *p* < 0.01) of DOX-treated rats, but this enhancement was largely normalized by ω-3 PUFAs pretreatment (Figure 7A,B; *p* < 0.01, *p* < 0.05, respectively). However, in our study, gene expression of anti-apoptotic factors (Bcl-2, Bcl-xl) were not statistically changed, except for the increased gene expression of Bcl-2 (Figure 7D, *p* < 0.05) in the hippocampus of rats pretreated with ω-3 PUFAs, and an increase in gene expression of Bcl-xl (Figure 7E, *p* < 0.05) in the prefrontal cortex of DOX-treated rats.

## 4. Discussion

The present study firstly demonstrated the protective role of ω-3 PUFAs against DOX-induced neurotoxicity in rats. We observed that DOX administration induced depressive-like behaviors in rats and pretreatment with ω-3 PUFAs normalized behavioral changes in rats treated with DOX. We also found that DOX caused oxidative stress, neuroinflammation, and cell death in the brain tissues and ω-3 PUFAs could partly alleviate these changes, suggesting the potentially protective role of ω-3 PUFAs in the brain from this pathophysiology. These findings are significant since patients following DOX chemotherapy are prone to develop depression and the findings in the present study raise the possibility that ω-3 PUFAs might be an adjuvant therapy and help to prevent this neurotoxic side effect of DOX in clinical practice.

Induction of depression-like behavior in rats using DOX has been mentioned in previous studies [3,27]. Our results indicated that DOX markedly promoted the depressive-like behaviors in rats, confirming the neurotoxical effect of DOX. In our preliminary work, a dose of 1.5 g/kg ω-3 PUFAs was effective to attenuate depressive behaviors in rats exposed to chronic unpredictable mild stress (CUMS) [15]. Similarly, in the present study, ω-3 PUFAs supplementation effectively restored these behavioral changes induced by DOX, showing robust antidepressant-like effects. However, it should be noted that although both EPA and DHA contain robust antidepressant properties [28,29,30], previous studies showed that EPA is more effective in mitigating the behavioral changes [30,31]. In this context, future studies are warranted to investigate which nutritional ingredient plays a major role in the neuroprotective effects of ω-3 PUFAs against DOX-induced behavioral changes.

According to the previous findings [11,13,15,32,33], the mechanisms underlying the behavioral changes following DOX treatment and the antidepressant-like and neuroprotective effects of ω-3 PUFAs might be related to the oxidative stress, inflammatory, and apoptotic status of the brain tissues. Thus, we further assessed the various markers of oxidative stress, inflammation, and apoptosis in different groups.

The oxidative stress as a plausible pathomechanism of neuropsychological alterations is strongly supported by previous findings [2,27], showing that DOX increases oxidative stress and reduces the total antioxidant capacity. In accordance with previous results, we observed that lipid peroxidation, a downstream chain reaction initiated by free radicals, was activated by DOX and the endogenous antioxidant enzyme SOD, responsible for scavenging superoxide radicals, was markedly suppressed by DOX in both prefrontal cortex and hippocampus, confirming the pro-oxidative effect of DOX on the brain tissues [34]. Furthermore, in this work, we clearly demonstrated the capability of ω-3 PUFAs in attenuating brain lipid peroxidation and protecting antioxidant enzyme activity in rats exposed to DOX, illustrating the effective anti-oxidative actions of ω-3 PUFAs against a potent free radical-producing chemotherapeutic agent. This is consistent with previous results when ω-3 PUFAs attenuated the oxidative damage to the heart or the testis of rats treated with DOX [17,18].

Moreover, previous studies have shown that ROS activates pro-inflammatory mediators, such as TNF-α and NF-κB, and subsequently induces brain neuroinflammation [35,36]. Pro-inflammatory cytokines such as IL-1β, IL-6, TNF-α, NF-κB, and iNOS have been demonstrated to induce abnormal behaviors, such as decreased locomotor activity, exploration, and depression [1,3,37], which was confirmed in our experiment. We found that DOX provoked generation of TNF-α and subsequently caused the activation of NF-κB and iNOS and increased the expression of genes required to control infection and injury, such as IL-1β and IL-6, indicating severe inflammatory conditions in the brain. Nevertheless, the current study elaborated the effective inhibition of generation of pro-inflammatory mediators by ω-3 PUFAs in brain tissues, including IL-1β, IL-6, TNF-α, NF-κB, and iNOS. This anti-inflammatory effect of ω-3 PUFAs has been reported elsewhere, when ω-3 PUFAs antagonize the NF-κB signaling pathway, and inhibit the expression of inflammatory genes downstream of NF-κB [12,38,39].

Additionally, we found that DOX caused a significant increase in TUNEL-positive neurocytes, suggesting severe DNA damage and neuronal death. Previous study conducted by Kreisel and his co-workers (2014) showed that CUMS-induced depression-like behavior in rodents exerted apoptotic cell death in hippocampus, indicating the close relationship between neural apoptosis and depression [40]. Therefore, apoptosis might play a causative role in the development of DOX-induced depression. As previous findings in rat cortical neurons [41], we also observed that the pro-apoptotic Bax was responsive to DOX stimulation in neurocytes, indicating the involvement of Bcl-2 family proteins in the pro-apoptotic effect of DOX. On the other hand, the present study elucidated the effective suppression of apoptosis and rescue of neurocytes by ω-3 PUFAs in brain tissues exposed to DOX. This finding implied that ω-3 PUFAs can block apoptosis, which is in accordance with previous research [12,42]. Although the mechanism responsible for the anti-apoptotic effect of ω-3 PUFAs was not yet defined, it might be partially associated with regulation of the expression of the Bcl-2 family of proteins, as gene expression of Bax in brain tissues was significantly inhibited by ω-3 PUFAs in our study. In line with our results, Paterniti *et al*. (2014) had demonstrated that the treatment with DHA reduced Bax expression in the brain tissues of mice [12]. Moreover, previous studies suggested that pro-inflammatory cytokines appear to contribute to depression-associated cell death through intrinsic apoptotic pathways and that neurotoxic free radicals are a second apoptosis-mediating factor associated with depressive disorder [12,20,43], suggesting that anti-oxidant and anti-inflammatory effects of ω-3 PUFAs could, in turn, indirectly contribute to its anti-apoptotic effect.

More importantly, there are a large number of studies reporting the anticancer and anti-cachectic effects of ω-3 PUFAs in a variety of model systems [44,45,46]. Eltweri *et al*. (2016) [45] provided evidence of the effectiveness of ω-3 PUFAs in cancer management with favorable outcomes, including better quality of life, less toxicity, and even improved survival, confirming the anti-cancer activity of ω-3 PUFAs. Additionally, Kim *et al*. (2016) [46] showed that the combination of regorafenib and DHA results in a synergistic effect upon tumor invasiveness and a reduction in tumor weights. These results indicate that adjunctive therapy with ω-3 PUFAs is more likely to enhance anti-tumor properties of DOX and reduce its neurotoxicity.

## 5. Conclusions

Collectively, we demonstrated that ω-3 PUFAs supplementation alleviated DOX-induced depressive-like behaviors and neurotoxicity in rats. The possible mechanisms underlying these behavioral-modulating and neuroprotective effects are proved to be at least partially associated with the anti-oxidant, anti-inflammatory, and anti-apoptotic actions of ω-3 PUFAs in brain tissues. Furthermore, this study provided us with a new potential treatment for brain damage induced by chemotherapeutic drugs, and had paved the way for further studies to investigate other mechanisms underlying the behavior modulating and neuroprotective effects of ω-3 PUFAs.

## Figures and Tables

**Table 1:** Primers used in real-time PCR analyses of mRNA expression.

Target Gene		Primers Sequences	Size (bp)
IL-1β	Forward	5′-AGGTCGTCATCATCCCACGAG-3′	119
	Reverse	5′-GCTGTGGCAGCTACCTATGTCTTG-3′	
IL-6	Forward	5′-CACAAGTCCGGAGAGGAGAC-3′	167
	Reverse	5′-ACAGTGCATCATCGCTGTTC-3′	
TNF-α	Forward	5′-GAGAGATTGGCTGCTGGAAC-3′	82
	Reverse	5′-TGGAGACCATGATGACCGTA-3′	
Bax	Forward	5′-CCAGGACGCATCCACCAAGAAGC-3′	135
	Reverse	5′-TGCCACACGGAAGAAGACCTCTCG-3′	
Bcl-xl	Forward	5′-CAGCTTCATATAACCCCAGGGAC-3′	207
	Reverse	5′-GCTCTAGGTGGTCATTCAGGTAGG-3′	
Bcl-2	Forward	5′-AGCCCTGTGCCACCTGTGGT-3′	93
	Reverse	5′-ACTGGACATCTCTGCAAAGTCGCG-3′	
β-Actin	Forward	5′-CATCCTGCGTCTGGACCTGG-3′	116
	Reverse	5′-TAATGTCACGCACGATTTCC-3′	
